# MRI-Derived Restriction Spectrum Imaging Cellularity Index is Associated with High Grade Prostate Cancer on Radical Prostatectomy Specimens

**DOI:** 10.3389/fonc.2015.00030

**Published:** 2015-02-17

**Authors:** Michael A. Liss, Nathan S. White, J. Kellogg Parsons, Natalie M. Schenker-Ahmed, Rebecca Rakow-Penner, Joshua M. Kuperman, Hauke Bartsch, Hyung W. Choi, Robert F. Mattrey, William G. Bradley, Ahmed Shabaik, Jiaoti Huang, Daniel J. A. Margolis, Steven S. Raman, Leonard S. Marks, Christopher J. Kane, Robert E. Reiter, Anders M. Dale, David S. Karow

**Affiliations:** ^1^Department of Urology, University of California San Diego School of Medicine, San Diego, CA, USA; ^2^Department of Radiology, University of California San Diego School of Medicine, San Diego, CA, USA; ^3^Department of Pathology, University of California San Diego School of Medicine, San Diego, CA, USA; ^4^Department of Pathology, University of California Los Angeles Geffen School of Medicine, Los Angeles, CA, USA; ^5^Department of Radiology, University of California Los Angeles Geffen School of Medicine, Los Angeles, CA, USA; ^6^Department of Urology, University of California Los Angeles Geffen School of Medicine, Los Angeles, CA, USA; ^7^Department of Neurosciences, University of California San Diego, La Jolla, CA, USA

**Keywords:** prostate, MRI imaging, prostate cancer, cellularity, Gleason score

## Abstract

**Purpose:** We evaluate a novel magnetic resonance imaging (MRI) technique to improve detection of aggressive prostate cancer (PCa).

**Materials and Methods:** We performed a retrospective analysis of pre-surgical prostate MRI scans using an advanced diffusion-weighted imaging technique called restriction spectrum imaging (RSI), which can be presented as a normalized *z*-score statistic. Scans were acquired prior to radical prostatectomy. Prostatectomy specimens were processed using whole-mount sectioning and regions of interest (ROIs) were drawn around individual PCa tumors. Corresponding ROIs were drawn on the MRI imaging and paired with ROIs in regions with no pathology. RSI *z*-score and conventional apparent diffusion coefficient (ADC) values were recorded for each ROI. Paired *t*-test, ANOVA, and logistic regression analyses were performed.

**Results:** We evaluated 28 patients with 64 ROIs (28 benign and 36 PCa). The mean difference in RSI *z*-score (PCa ROI–Benign ROI) was 2.17 (SE = 0.11; *p* < 0.001) and in ADC was 551 mm^2^/s (SE = 80 mm^2^/s; paired *t*-test, *p* < 0.001). The differences in the means among all groups (benign, primary Gleason 3, and primary Gleason 4) was significant for both RSI *z*-score (*F*_3,64_ = 97.7, *p* < 0.001) and ADC (*F*_3,64_ = 13.9, *p* < 0.001). A *t*-test was performed on only PCa tumor ROIs (*n* = 36) to determine PCa aggressiveness (Gleason 3 vs. Gleason 4) revealing that RSI *z*-score was still significant (*p* = 0.03), whereas, ADC values were no longer significant (*p* = 0.08). In multivariable analysis adjusting for age and race, RSI *z*-score was associated with PCa aggressiveness (OR 10.3, 95% CI: 1.4–78.0, *p* = 0.02) while ADC trended to significance (*p* = 0.07).

**Conclusion:** The RSI-derived normalized cellularity index is associated with aggressive PCa as determined by pathologic Gleason scores. Further utilization of RSI techniques may serve to enhance standardized reporting systems for PCa in the future.

## Introduction

One current focus in prostate cancer (PCa) diagnosis is to distinguish indolent from more aggressive disease to reduce over-treatment (1, 2). Magnetic resonance imaging (MRI) may be a non-invasive imaging biomarker to incorporate into PCa detection and treatment strategies (3).

Recently, MRI has been increasingly investigated for use as a tool in the screening, staging, and monitoring of PCa (4–6). Imaging techniques such as diffusion-weighted imaging (DWI) and the resultant quantitative apparent diffusion coefficient (ADC) have shown correlation with PCa; however, they have significant limitations regarding tumor conspicuity and localization (7, 8). A novel, advanced diffusion-based imaging technique, called restriction spectrum imaging (RSI), has been modified from previous studies in brain cancer detection to apply to patients with PCa (8, 9). Some benefits of the RSI technique include reduced spatial distortion, enhanced tumor contrast-to-noise over conventional diffusion-weighted imaging (DWI), and a normalized *in vivo* measure of cellularity.

Within individual tumor regions of interest (ROIs), we investigate the association of final pathologic Gleason score from whole-mount prostatectomy specimens with the RSI cellularity index as compared to the current standard, ADC (10–16). Our primary outcome is the detection of primary pattern Gleason ≥4 PCa.

## Materials and Methods

### Patients

All patients were previously diagnosed with PCa via standard transrectal ultrasound guided prostate biopsy after prostate specific antigen (PSA) elevation or abnormal digital rectal examination (DRE). Pre-surgical pelvic MRI is routinely used at our institution to identify extraprostatic extension (EPE) in order to guide nerve-sparing surgery. Data were collected from chart review.

### MRI and RSI

Patients underwent standard T2, perfusion (with Gadolinium) and diffusion protocols at 3 T (Siemens, Erlangen Germany) with an endorectal coil prior to radical prostatectomy. Table 1 shows pulse sequence details. A modified Prostate Imaging-Reporting and Data System (PIRADS) score, termed “the UCLA assessment criteria,” was assigned to the scan based on the suspicion of cancer previously instituted at our institution (17). The ADC maps used to draw ROIs were generated from the low *b*-value, 800 s/mm^2^, derived from the same spectrum of *b*-values used in the RSI protocol. ADC maps were corrected for spatial distortion (18). The restriction spectrum diffusion tensor imaging protocol parameters include *b*-values of 0, 800, 1500, 4000 s/mm^2^ in 30 unique diffusion directions for each non-zero *b*-value. RSI cellularity maps were reconstructed based on all *b*-values (8). The RSI cellularity maps were then standardized across the sample, using the mean and standard deviations of normal prostate signal from the raw RSI maps in 20 patients to produce *z*-score maps. RSI maps were also corrected for spatial distortion (19).

**Table 1:** **MRI scan parameters for prostate MRI protocol at 3T**.

Pulse sequence	Parameters
T2	Axial 3D TSE T2 (Siemens SPACE), TR/TE 3800-5040/101, ETL 13, 14 cm FOV, 256 × 256 matrix, 1.5 mm contiguous slices
Diffusion-weighted (standard)	echoplanar, TR/TE 3900/60, 21 × 26 cm FOV, 130 × 160 matrix, 3.6 mm slices, 4 NEX, *b*-values 0, 100, 400, 800 s/mm^2^
T1 dynamic perfusion imaging	Siemens TWIST, TR/TE 3.9/1.4 ms, 12° flip angle, 26 × 26 cm FOV, 160 × 160 matrix, 3.6 mm slices, 4.75 s/acquisition over 6 minutes with 15 s injection delay, image analysis using iCAD Versavue
Restriction spectrum imaging	Spin echo EPI, TR/TE 5500/137, 26 cm × 26 cm FOV, 128 × 96 matrix, 3.6 mm slices, 30 directions at each *b*-value, *b*-values 0, 800, 1500, 4000 s/mm^2^

### Pathology

After prostatectomy, whole-mount histopathology was routinely performed on 4 μm thick sections of each specimen. A Gleason score was assigned to each representative tumor location. If two tumors were located, the Gleason score for each was assessed independently. The histopathology was evaluated and the boundaries of tumor vs. benign tissue were identified by an uropathologist.

### Outcomes

We defined our primary outcome as pathologic primary Gleason score of 4, which means that Gleason 4 is the dominant histologic architecture and includes 4 + 3, 4 + 4, and 4 + 5 Gleason patterns. The pathologic Gleason score is currently the standard of reference for PCa aggressiveness. Additionally, the ability of imaging to detect secondary Gleason patterns may be minimal; therefore, herein we focus on primary Gleason patterns. Our primary predictor variable was the normalized cellularity index called the “RSI *z*-score.” The most commonly utilized tool to identify and classify aggressive cancer on MRI currently is the ADC value from DWI; therefore, the RSI *z*-score was compared with ADC to assess the predictive value in differentiating cancer from normal ROI.

Each patient had at least one identified region of cancerous tissue. If two areas of cancer were detected, each region was evaluated and assigned a separate Gleason score, ADC, and *z*-score. Tumor ROIs were drawn based on the pathology in combination with ADC images that had been corrected for spatial distortion. A benign ROI was defined in a region of the prostate seen to be free of PCa on the whole-mount histology. ADC and RSI *z*-score values were recorded for all ROIs.

### Statistical analysis

Each patient had at least one ROI of cancer and one ROI of benign tissue. Correlation between the RSI-derived *z*-score and ADC was determined by a Pearson correlation test assuming normal distribution. In order to investigate the association of RSI *z*-score and primary Gleason pattern 4 PCa vs. pattern 3 PCa, a *t*-test was performed. In order to compare the utility of MRI techniques (RSI *z*-score vs. ADC) for detecting aggressive cancer, we compared ROIs representing pathologically benign tissue with those representing increasing aggressive PCa (benign vs. Gleason 3 vs. Gleason 4 primary patterns) using ANOVA analysis (*F*-test). After removing the values for the benign ROIs, we also assessed variation in MRI values among different grades of cancerous tissue aggressiveness by performing a comparative *t*-test. Multivariable analysis included an ordinal logistic regression (benign vs. Gleason 3 vs. Gleason 4) and binary logistic regression (Gleason 3 vs. Gleason 4). *p*-values <0.05 were considered statistically significant using the statistical package SPSS v.21 (IBM, Chicago, IL, USA). Age and race were controlled for due to the risk of PCa associated with these variables and that they are inherent to each ROIs. However, other demographic variables associated with cancer (PSA, clinical stage, biopsy data, etc.) may not be associated with an individual ROI and may misrepresent the data as some patients have multiple ROIs. Therefore, the multivariable analysis only includes the preselected variables of age and race without accounting for these other variables despite their significance in univariable analysis.

## Results

**Figure 1:**
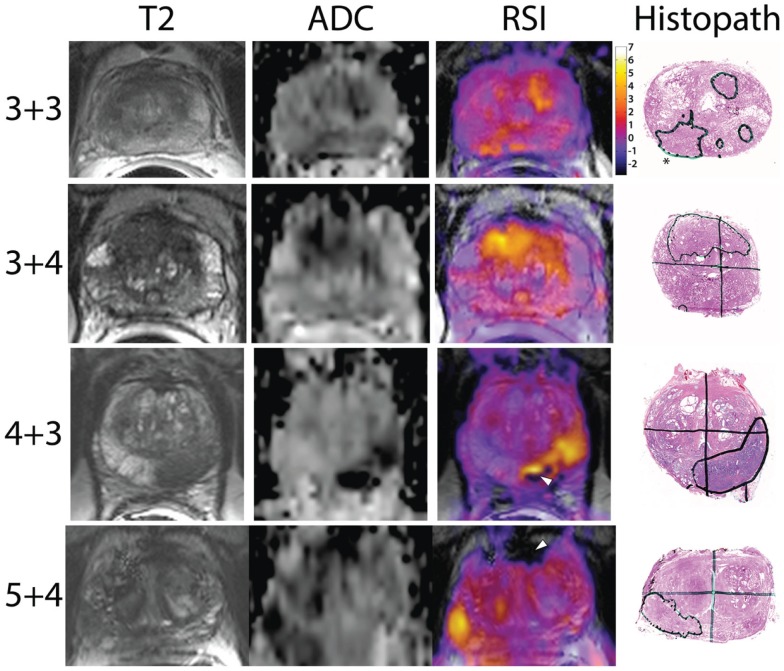
**Representative images showing RSI *z*-score maps across Gleason scores: the *y*-axis shows the pathologic Gleason score with the *x*-axis designating the MRI sequence**. The last column displays the whole-mount pathology with the corresponding cancer region of interest circled in black. The star in the top right pathologic figure represents the pattern 3 + 3 prostate cancer while the other lesions are 4 + 3. White arrowheads in the higher-grade patients show areas of signal void, which could be interpreted as false positives on the ADC maps.

**Figure 2:**
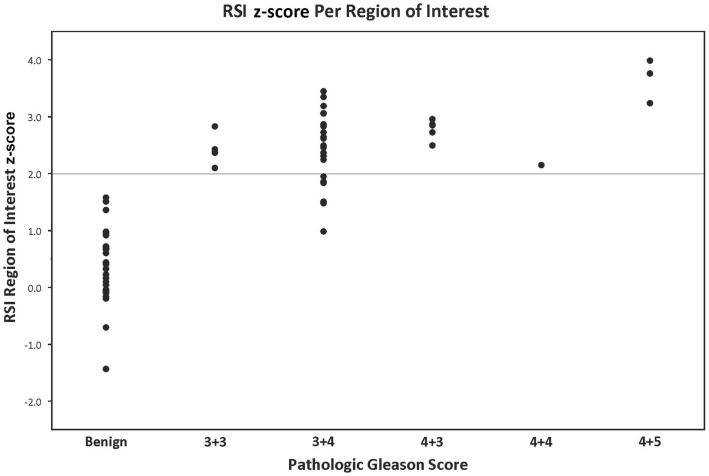
**RSI *z*-score value grouped by pathologic Gleason score: the *y*-axis represents the RSI *z*-score derived from a given region of interest**. The *x*-axis demonstrates the pathological Gleason scores in increasing levels of aggressiveness from left to right. Each data point represents one region of interest corresponding to a location on the whole-mount prostatectomy specimen contoured by a GU pathologist.

After IRB approval (UCLA IRB#12-001301), we identified 28 patients who underwent preoperative MRI with RSI and subsequent whole-mount pathology after radical prostatectomy, with surgery taking place between May 2012 and May 2013. Demographics are displayed in Table 2. Figure 1 shows representative examples of RSI across the Gleason spectrum and illustrates data collection methods. White arrows within the higher-grade RSI maps show areas of signal void that could be interpreted as false positives on the corresponding ADC maps. We identified 64 ROIs (28 benign and 36 PCa). Eight patients had two distinct tumor ROIs within one specimen. Seven of those patients had discordant tumors (two different Gleason scores) and only one patient had two concordant tumor ROIs (both Gleason 3 + 4). The RSI *z*-score data for all ROIs, grouped by Gleason score, is shown in Figure 2.

**Table 2:** **Demographics: perioperative demographics for 28 patients who underwent MRI with an endorectal coil and subsequently underwent radical prostatectomy**.

Age	Race	BMI	PSA	Clinical Stage	Biopsy Gleason	Positive cores	Maximum percent cancer %	Imaging criteria	Pathologic Gleason	Pathologic stage
63	White	20.1	7	T1c	3 + 4	3 of 15	30	4	3 + 3	T2
61	White	31.4	5.8	T1c	4 + 3	4 of 12	85	5	3 + 4	T3b
55	White	29.3	2.8	T1c	3 + 5	4 of 12	90	3	3 + 4	T3a
71	White	29.7	7.3	T2	3 + 3	9 of 19	60	3	3 + 4	T2
59	White	31.4	4.43	T2	3 + 3	4 of 12	33	3	3 + 4	T2c
61	White	21.4	9.2	T2	3 + 3	6 of 15	30	4	3 + 4	T2b
68	White	–	6.7	T1c	3 + 4	10 of 12	70	3	3 + 4	T2c
55	White	27.5	4.7	T1c	3 + 4	6 of 16	70	3	3 + 4	T2b
64	White	23.1	5.8	T1c	3 + 4	1 of 12	30	2	3 + 4	T2c
61	Other	26	3.4	T1c	3 + 4	6 of 13	95	3	3 + 4	T2c
60	Unknown	25.5	6	T1c	3 + 3	3 of 12	–	3	3 + 4	T2
61	White	29.8	6.6	T1c	–	–	–	4	3 + 4	T2
65	Unknown	29	5.4	T2	3 + 4	2 of 14	45	2	3 + 4	T2
50	White	31.2	8.9	T1c	3 + 4	2 of 12	20	4	3 + 4	T2
53	White	22.7	3.9	T1c	4 + 3	3 of 8	15	2	3 + 4	T3a
44	White	26.6	2.8	T1c	3 + 4	4 of 16	70	3	3 + 4	T2a
58	White	32.7	3.8	T1c	3 + 4	6 of 12	70	3	3 + 4	T2c
53	Asian	16.9	5.5	T1c	3 + 4	3 of 12	15	4	3 + 4	T2
62	African American	25.1	4.8	T1c	4 + 3	2 of 12	70	0	4 + 3	T2a
65	White	38.5	5.8	T2	4 + 3	5 of 12	88	5	4 + 3	T3a
64	White	26.9	4.2	T1c	3 + 5	12 of 12	94	5	4 + 3	T3a
56	Other	28	11.5	T1c	3 + 4	multiple	50	4	4 + 3	T3a
67	White	30.4	5.1	T1c	3 + 4	5 of 13	25	4	4 + 3	T3a
71	White	28.4	12.8	T1c	4 + 3	–	–	5	4 + 3	T3a
45	African American	23.5	6.5	T1c	4 + 3	9 of 16	80	1	4 + 4	T2c
65	White	30.1	8.2	T1c	5 + 4	–	55	4	4 + 5	T3b
62	White	25.8	4.6	T2	4 + 5	5 of 15	50	4	4 + 5	T2
70	Filipino	35.5	7.5	T2	4 + 3	9 of 12	90	3	4 + 5	T3a

Apparent diffusion coefficient and RSI *z*-scores are normally distributed and did not need further transformation. There is a significant correlation between ADC and RSI *z*-score (Pearson *R* = 0.69; *n* = 64, *p* < 0.001) (see supplementary figure). ADC and RSI *z*-score are collinear (collinearity index 8.4); therefore, they cannot be placed within the same multivariate analytic model.

The mean cellularity index (RSI *z*-score) in PCa was 2.53 (SE = 0.10) and in benign tissue 0.39 (SE = 0.12; *p* < 0.001). The mean ADC for PCa was 1169 mm^2^/s (SE = 67 mm^2^/s) and benign was 1679 mm^2^/s (SE = 81 mm^2^/s, *p* < 0.001).

**Figure 3:**
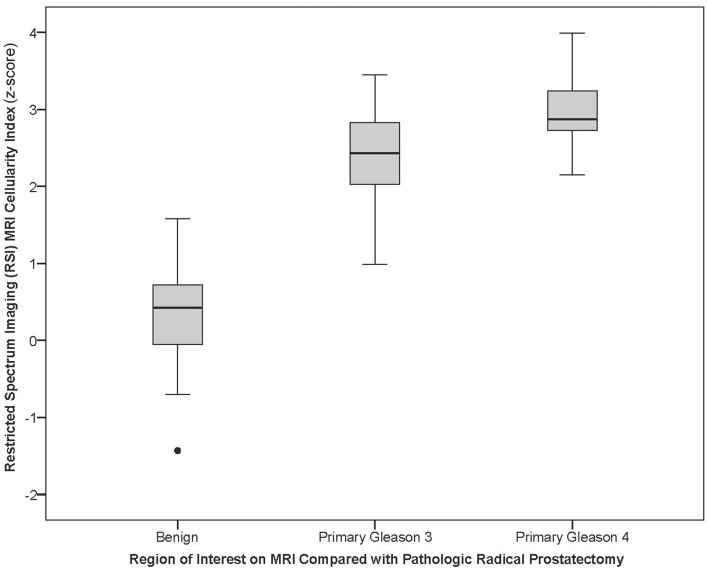
**Box plot of RSI *z*-score for primary Gleason pattern: the box plot represents the RSI *z*-score for benign, pathologic primary pattern Gleason 3, or pathologic primary pattern Gleason 4 prostate cancer**.

For the univariate and multivariate analyses, the data were grouped by primary Gleason pattern, either primary Gleason 3 (3 + 3 or 3 + 4) or Gleason 4 (4 + 3, 4 + 4, or 4 + 5). In univariable analysis, we investigate the association of RSI *z*-score and ADC with PCa on pathology (benign vs. Gleason 3 vs. Gleason 4) using ANOVA analysis. Both were able to distinguish benign from increasingly malignant PCa (both *p* < 0.001) (Table 3). Figure 3 displays a box plot developed from individual ROI RSI *z*-scores corresponding to benign tissue primary Gleason 3 pattern or primary Gleason 4 pattern PCa tumors. When removing the benign ROIs as to only compare low-grade (primary Gleason 3) to high-grade PCA (primary Gleason 4), RSI technique was able to distinguish the two groups (*p* = 0.03) and ADC trended to significance (*p* = 0.08). We then investigated these same parameters in multivariable analysis and determined that both RSI *z*-score and ADC were able to distinguish between the three groups in ordinal regression analysis adjusting for age and race (white vs. non-white); though the log odds of the parameter estimates suggest an improved distinction of the groups by RSI (RSI *z*-score and ADC, *p* < 0.001). However, when determining the difference in the detection of low-grade and high-grade PCa, a higher RSI *z*-score was significantly associated with the higher-grade primary Gleason 4 pattern [Odds ratio 10.3 (1.4–78.0; *p* = 0.02)] and ADC showed a trend in distinguishing between the two Gleason patterns (*p* = 0.07) (Table 4).

**Table 3:** **Univariable analysis: restriction spectrum imaging (RSI) *z*-score and apparent diffusion coefficient (ADC) are independently investigated comparing individual regions of interest to their corresponding primary Gleason pattern**.

Sample size method			Means	Statistical test	*p* value

**Detection of increasingly aggressive cancer^a^**			Mean (standard error)	*F*-test	
64	Restricted spectrum imaging (RSI) *z*-score			97.7	<0.001
		Benign	0.65 (0.12)		
		Primary Gleason 3	2.4 (0.61)		
		Primary Gleason 4	2.9 (0.51)		
64	Apparent diffusion coefficient (ADC)			13.9	<0.001
		Benign	1680 (428)		
		Primary Gleason 3	1237 (425)		
		Primary Gleason 4	967 (221)		

**Prostate cancer aggressiveness^b^**			**Mean difference**	***t*-test**	

36	Restricted spectrum imaging (RSI) *z*-score	34	0.5	2.22	0.033
36	Apparent diffusion coefficient (ADC)	34	269	1.81	0.079

*The top half of the table uses ANVOA analysis to determine differences in detection comparing benign vs. primary Gleason 3 vs. Primary Gleason 4 prostate cancer. The bottom half of the table only compares Gleason 3 vs. 4 cancers with a *t*-test*.

*^a^Benign vs. Gleason 3 vs. Gleason 4*.

*^b^Gleason 3 vs. Gleason 4*.

**Table 4:** **Multivariable analysis: restriction spectrum imaging (RSI) *z*-score and apparent diffusion coefficient (ADC) are independently investigated comparing individual regions of interest to their corresponding primary Gleason pattern**.

Sample size method			Parameter coefficient^a^ (95% confidence interval)	*p* value	McFadden’s *D*

**Detection of increasingly aggressive prostate cancer^b^**					
64	Restricted spectrum imaging (RSI) *z*-score			<0.001	0.613
		Benign	0		
		Primary Gleason 3	9.5 (−1.9 to 17.0)	0.014	
		Primary Gleason 4	15.8 (6.4–25.2)	0.001	
64	Apparent diffusion coefficient (ADC)			<0.001	0.202
		Benign	0		
		Primary Gleason 3	1.7 (−3.2 to 6.7)	0.483	
		Primary Gleason 4	4.5 (−0.5 to 9.6)	0.076	

**Prostate cancer aggressiveness^c^**		**Wald test**	**Odds ratio (95% confidence interval)**	***p* value**	

36	Restricted spectrum imaging (RSI) *z*-score	5.1	10.3 (1.4–78.0)	0.024	
36	Apparent diffusion coefficient (ADC)	3.3	0.9 (0.9–1.00)	0.069	

*The top half of the table uses ordinal logistic regression analysis to determine differences in detection comparing benign vs. primary Gleason 3 vs. Primary Gleason 4 prostate cancer (Log Odds). The bottom half of the table only compares Gleason 3 vs. 4 cancers to determine with binary logistic regression (Odds Ratio)*.

*^a^Parameter coefficients are the log odds and can be converted to odds ratios by taking the exponent of the estimate. The numbers are too large therefore are kept as log odds for simplicity*.

*^b^Ordinal logistic regression (benign vs. Gleason 3 vs. Gleason 4) adjusting for age and race (White vs. Non-White)*.

*^c^Binary logistic regression (Gleason 3 vs. Gleason 4) adjusting for age and race (White vs. Non-White)*.

## Discussion

Magnetic Resonance RSI normalized cellularity index (RSI *z*-score) is able to distinguish aggressive PCa (primary Gleason score of 4 compared to 3) in our population of men undergoing radical prostatectomy. Importantly, the RSI technique has displayed at least similar ability to distinguish Gleason grade to the current reference standard, ADC values.

Multiple studies have described the ability of ADC to detect PCa. However, the distinction between PCa aggressiveness has been less investigated. Donati et al. found that mean ADC could distinguish Gleason 6 from 7+ tumors in 131 men undergoing prostatectomy (AUC 0.706) and in another paper discussed the use of 10^th^ percentile ADC correlation to aggressiveness (10, 16). The 10^th^ percentile ACD was also used in combination with mean ADC, T2-weighted skewness, and *K*^trans^ to distinguish PCa using computer aided diagnosis (18). Moreover, a recent study has suggested that ADC entropy rather than mean ADC could better discriminate the proportion of Gleason 4 cancer among Gleason 3 + 4 and 4 + 3 tumors (20). The distinction in primary Gleason pattern may have significant clinical implications regarding PCa management decision-making.

Primary Gleason 4 pattern is a more aggressive cancer with patients experiencing higher rates of biochemical (PSA) failure after prostatectomy, systemic recurrence, and PCa mortality (21). Therefore, knowledge of high-grade cancer prior to making management decisions would be helpful in determining treatment strategy. For example, men without Gleason 4 pattern PCa are more ideal candidates for active surveillance (22). Serial imaging may indicate progression of disease, assisting urologists in deciding when a biopsy-off of protocol is warranted. Additionally, PCa grade may influence the urologic surgeon to perform a pelvic lymph node dissection at the time of prostatectomy due to increased risk of nodal disease (23).

Currently, ADC serves as the most discriminatory parameter to assist radiologists for the detection of cancer. Moreover, recent examination of ADC and PCa has shown the association of ADC and PCa aggressiveness (10, 16). While we do show that ADC can differentiate the presence of cancer or not, our study shows that ADC is less able to determine the subtlety of primary pattern Gleason 3 vs. Gleason 4 PCa. Possible reasons include the proportion of pattern 3 vs. 4 disease in our population compared to prior populations, the *b*-values used in determining the ADC, how the ROI was chosen, the amount of stromal reaction, and technical factors such as degree of hemorrhage. However, the differences between ADC and RSI *z*-score in our study are small.

Restriction spectrum imaging techniques offer advantages when compared to conventional DWI and ADC maps. For example, one of the challenges of standard diffusion imaging is that the ADC values are not standardized across MRI scanners. The *z*-score is a standardized statistical method and inherently normalizes across the patient pool. Thus, the RSI *z*-score is a value that could potentially be compared across different scanners and institutions and provide a more robust value for relative comparison.

In addition, conventional DWI/ADC suffers from geometric distortion and can be difficult to interpret by clinicians and untrained radiologists. Distortion correction techniques previously optimized in the brain for GBM, are incorporated into our RSI post-processing stream in order to derive spatially corrected cellularity maps. Distortion correction techniques are not routinely employed in conventional DWI and resultant ADC maps. Thus, the RSI maps can be co-registered with T2-weighted anatomic images with voxel accuracy. This has potential implications for more accurate detection of EPE and more accurate MRI-fused ultrasound targeted biopsy results (24).

Because of its greater sensitivity to restricted rather than hindered diffusion, RSI may be less subject to hemorrhage, inflammatory processes, and benign nodules in the transitional zone, all of which can exhibit lower ADC values leading to false positives. Theoretically, RSI-MRI reduces extracellular signal by focusing on the signal emanating from intracellular tumor cells (restricted diffusion) and less from the extracellular signal (hindered diffusion) (8, 9). This will need to be rigorously tested in future ROC performance studies.

Apparent diffusion coefficient maps will exhibit low signal in regions where there is overt chemical dephasing from gross calcium, hemorrhage, or other etiologies resulting in signal void. Unfortunately, these signal voids could be interpreted inaccurately, leading to a false positive result. White arrow heads in Figure 1 show two such examples. For example, in the Gleason 5 + 4 case, the anterior region of signal void shows up as dark (low) on the ADC maps, potentially a false positive, while in the RSI maps, this is clearly interpreted as an area of signal void, not tumor. Thus, RSI offers a number of potential advantages when compared to DWI/ADC.

Certain limitations of this study include small sample size and retrospective data collection. We have overcome the small sample size by using each patient as his own control to provide a paired analysis by using known benign tissue. However, because of the small sample size, we have fewer patients with extremely high-grade cancer (Gleason 5) and low-grade (Gleason 6 or less); therefore, we dichotomized based on the primary Gleason pattern. We justify this analysis by assuming that MRI imaging is unlikely to visualize smaller amounts (secondary patterns) of PCa architecture. The sample size was too small to evaluate upgrading or downgrading Gleason scores at prostatectomy from the initial biopsy results in order to determine if RSI could serve to differentiate these cases; however, this question will serve as a focus in future studies. We do have a selection bias regarding our patient population as all patients underwent radical prostatectomy. Therefore, our results may not necessarily apply to patients in the general PSA screening population undergoing prostate biopsy. The lack of patients in this study with pathologies at the extremes shows the need for a broader study.

## Conclusion

Restriction Spectrum Imaging cellularity index is associated with the detection of aggressive PCa as defined by Gleason score. Additionally, RSI-MRI includes correction of spatial distortion, a normalized measure of cellularity, and in general increased conspicuity when compared to conventional DWI/ADC. RSI technology warrants prospective evaluation in the PCa diagnostic arena.

## Conflict of Interest Statement

The authors declare that the research was conducted in the absence of any commercial or financial relationships that could be construed as a potential conflict of interest.

## Supplementary Material

The Supplementary Material for this article can be found online at http://www.frontiersin.org/Journal/10.3389/fonc.2015.00030/abstract
