# Supplementary material for: MRI-Derived Restriction Spectrum Imaging Cellularity Index is Associated with High Grade Prostate Cancer on Radical Prostatectomy Specimens
Source: Front Oncol. 2015 Feb 17;5:30. doi: 10.3389/fonc.2015.00030 (PMC4330697; doi:10.3389/fonc.2015.00030)
Supplement: Supplementary file 1 [file Image_1.PDF]

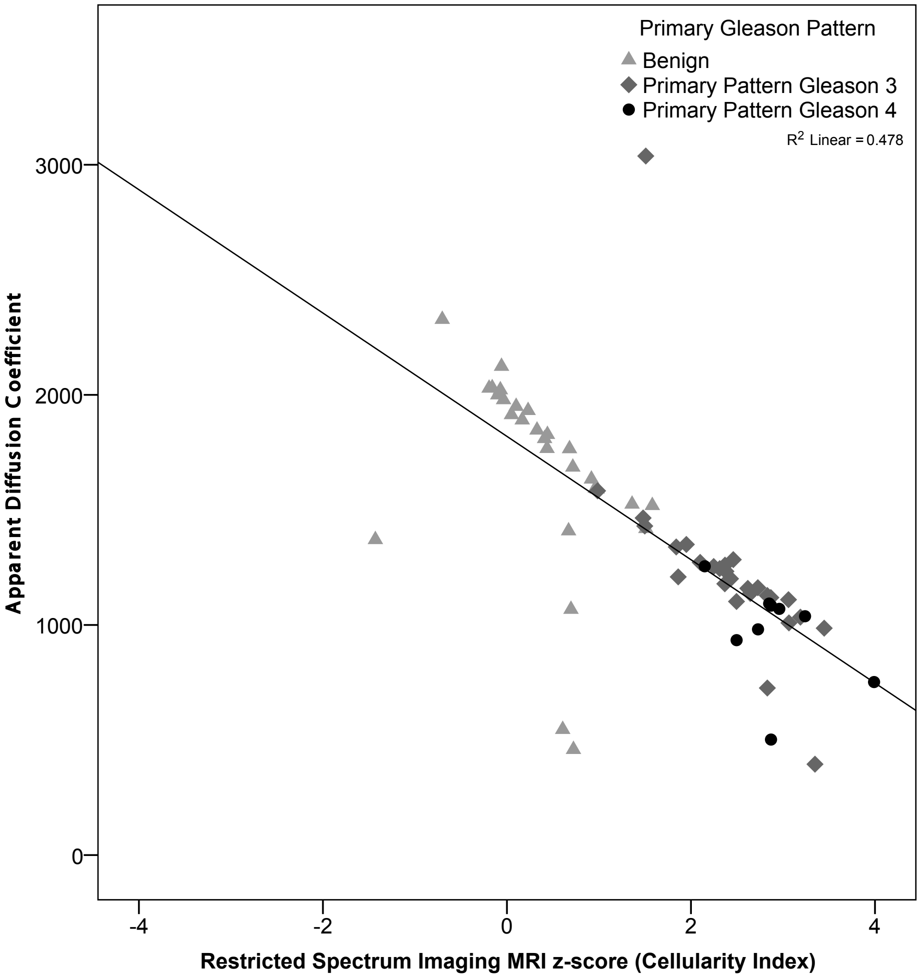

Supplementary Figure: Correlation of Apparent Diffusion Coefficient (ADC) and Restricted Spectrum Imaging (RSI) z-score. The scatter plot demonstrates a linear relationship between ADC and RSI in regards to the region of interest based on pathology results.
